# A multicentre randomised controlled trial of levetiracetam versus phenytoin for convulsive status epilepticus in children (protocol): Convulsive Status Epilepticus Paediatric Trial (ConSEPT) - a PREDICT study

**DOI:** 10.1186/s12887-017-0887-8

**Published:** 2017-06-22

**Authors:** Stuart R. Dalziel, Jeremy Furyk, Megan Bonisch, Ed Oakley, Meredith Borland, Jocelyn Neutze, Susan Donath, Cynthia Sharpe, Simon Harvey, Andrew Davidson, Simon Craig, Natalie Phillips, Shane George, Arjun Rao, Nicholas Cheng, Michael Zhang, Kam Sinn, Amit Kochar, Christine Brabyn, Franz E. Babl, On Behalf of the PREDICT research network

**Affiliations:** 1 Starship Children’s Hospital, Private Bag 92024, Auckland, 1142 New Zealand; 2 Liggins Institute, University of Auckland, Auckland, New Zealand; 3 The Townsville Hospital, Townsville, Queensland Australia; 4 James Cook University, Townsville, Queensland Australia; 5 Royal Children’s Hospital, Melbourne, Victoria Australia; 6 Murdoch Childrens Research Institute, Victoria, Australia; 7 Department of Paediatrics, University of Melbourne, Victoria, Australia; 8 Princess Margaret Hospital, Perth, Western Australia Australia; 9 Kidz First Hospital, Auckland, New Zealand; 10 Monash Medical Centre, Victoria, Australia; 11 Lady Cilento Children’s Hospital, Brisbane, Queensland Australia; 12 Gold Coast University Hospital, Southport, Queensland Australia; 13 University of Queensland, Brisbane, Queensland Australia; 14 Bond University, Gold Coast, Queensland Australia; 15 Sydney Children’s Hospital, Randwick, New South Wales Australia; 16 Children’s Hospital at Westmead, Sydney, New South Wales Australia; 17 John Hunter Hospital, Newcastle, New South Wales Australia; 18 Canberra Hospital, Canberra, Australian Capital Territory Australia; 19 Women’s and Children’s Hospital, Adelaide, South Australia Australia; 20 Waikato Hospital, Hamilton, New Zealand; 21 Paediatric Research in Emergency Departments International Collaborative, Melbourne, Victoria Australia

**Keywords:** Convulsive status epilepticus, Paediatrics, Emergency medicine, Levetiracetam, Phenytoin, Intervention study, Randomised controlled trial

## Abstract

**Background:**

Convulsive status epilepticus (CSE) is the most common life-threatening childhood neurological emergency. Despite this, there is a lack of high quality evidence supporting medication use after first line benzodiazepines, with current treatment protocols based solely on non-experimental evidence and expert opinion. The current standard of care, phenytoin, is only 60% effective, and associated with considerable adverse effects. A newer anti-convulsant, levetiracetam, can be given faster, is potentially more efficacious, with a more tolerable side effect profile. The primary aim of the study presented in this protocol is to determine whether intravenous (IV) levetiracetam or IV phenytoin is the better second line treatment for the emergency management of CSE in children.

**Methods/Design:**

200 children aged between 3 months and 16 years presenting to 13 emergency departments in Australia and New Zealand with CSE, that has failed to stop with first line benzodiazepines, will be enrolled into this multicentre open randomised controlled trial. Participants will be randomised to 40 mg/kg IV levetiracetam infusion over 5 min or 20 mg/kg IV phenytoin infusion over 20 min. The primary outcome for the study is clinical cessation of seizure activity five minutes following the completion of the infusion of the study medication. Blinded confirmation of the primary outcome will occur with the primary outcome assessment being video recorded and assessed by a primary outcome assessment team blinded to treatment allocation. Secondary outcomes include: Clinical cessation of seizure activity at two hours; Time to clinical seizure cessation; Need for rapid sequence induction; Intensive care unit (ICU) admission; Serious adverse events; Length of Hospital/ICU stay; Health care costs; Seizure status/death at one-month post discharge.

**Discussion:**

This paper presents the background, rationale, and design for a randomised controlled trial comparing levetiracetam to phenytoin in children presenting with CSE in whom benzodiazepines have failed. This study will provide the first high quality evidence for management of paediatric CSE post first-line benzodiazepines.

**Trial registration:**

Prospectively registered with the Australian and New Zealand Clinical Trial Registry (ANZCTR): ACTRN12615000129583 (11/2/2015). UTN U1111–1144-5272. ConSEPT protocol version 4 (12/12/2014).

## Background

Convulsive status epilepticus (CSE) is the most common life-threatening childhood neurological emergency [1]. It has an annual incidence of 17–23 cases per 100,000 children per year, with 22% of patients requiring Rapid Sequence Induction (RSI) and Intensive Care Unit (ICU) admission [2]. Mortality following paediatric CSE is reported at 3–5% and neurological sequelae occur in up to 34% of children [3].

Management guidelines for paediatric CSE recommend early and prompt use of anticonvulsant medication [4, 5]. Recommendations from the Advanced Life Support Group [4], the Scottish Intercollegiate Guidelines Network [5], the Status Epilepsy Working Party in the United Kingdom [6], and major textbooks [7, 8], are broadly similar and universally adopt a stepwise approach to treatment; 1.Two doses of benzodiazepine; 2.Second line anticonvulsant, with all recommending phenytoin or fosphenytoin; and 3.Final termination of CSE with RSI intubation with thiopentone and ICU admission. While there is reasonable evidence to support the use of benzodiazepines in CSE there is a paucity of evidence concerning the type and efficacy of second line anticonvulsant medication used with management guidelines based only on expert opinion [4, 5, 9].

The aetiology and outcomes of CSE in children is different to that of adults, thus adult evidence cannot be expected to be directly applicable to paediatric practice. A large population based study of CSE reported that less than a quarter of the children had a previous history of CSE. Of those with a first presentation of CSE over half were previously neurologically normal, a third of episodes were due to prolonged febrile convulsions, 17% of episodes were due to central nervous system (CNS) infection or acute metabolic derangement, with the remainder of episodes idiopathic or associated with a pre-existing CNS abnormality [2].

The term CSE was traditionally defined as 30 min of a continuous generalised tonic-clonic convulsion or recurrent tonic-clonic convulsions without recovery of consciousness between each convulsion [10]. Recently a revised operational definition based on the indication to commence treatment has defined CSE as seizures of a duration of five minutes or more [11]. This shortening of seizure duration for CSE definition is due to evidence that the natural history for typical generalised convulsive seizures is to resolve spontaneously by 3–5 min, with those not doing so requiring medication for termination [11, 12]. This revised definition has been adopted by recent trials on paediatric CSE [13]. Early medication use and cessation of seizures in CSE is important. There is a wealth of animal evidence suggesting that longer seizures are harmful and result in irreversible brain damage and poorer outcomes [1].

A survey of attending Paediatric Emergency Physicians in Australia and New Zealand confirmed that benzodiazepines are universally recommended for first line treatment in CSE and that 88% would use phenytoin as a second line agent, in keeping with guideline recommendations. However there was a large variation in third line agents, reflecting that the majority of consultants (68%) would try another agent prior to RSI [14]. A retrospective review of CSE management at eight large paediatric emergency departments (EDs) in New Zealand and Australia over five years identified 542 patients with CSE and found phenytoin resulted in cessation of seizures in only 60% of the 315 patients who received it as a second line anticonvulsant for CSE [15]. This success rate is comparable with other reported series [15–17].

In addition to its less than optimal effectiveness, phenytoin has a number of features that make it less than ideal to be used in CSE. Phenytoin is a potent inducer of hepatic enzymes resulting in reduced levels of a number of other anticonvulsants and non-anticonvulsant drugs. Its adverse events include hepatotoxicity, pancytopenia and Stevens-Johnson-Syndrome. Phenytoin can cause cardiac arrhythmias, hypotension, phlebitis, and severe soft tissue injury from extravasation and purple glove syndrome. Because of its cardiotoxicity it has to be given slowly (1 mg/kg/min) [16, 18]. Furthermore, phenytoin cannot be mixed with dextrose, a common component of paediatric intravenous (IV) fluids [16].

In North America fosphenytoin, the prodrug of phenytoin, is increasingly used instead of phenytoin [8, 16]. Although fosphenytoin can be administered more rapidly, the additional requirement to be metabolised into the active phenytoin means that it does not offer any true time advantages over phenytoin. However, fosphenytoin has a number of other advantages such as the ability to be administered intramuscularly and decreased infusion related adverse events, although deaths due to fosphenytoin infusions have been reported. Furthermore, there are no data to show that fosphenytoin is more effective than phenytoin in stopping seizures. Importantly, for the purposes of this study, fosphenytoin is neither available nor approved for use in New Zealand and Australia.

Newer antiepileptic drugs such as levetiracetam, valproate and lacosamide have been proposed [9, 16, 17], and have been reported as effective in case reports and small case series in adults and in children [19–22]. The most promising, Levetiracetam, a broad spectrum, antiepileptic drug has been approved for use for over a decade and is widely used internationally for maintenance seizure prophylaxis for both focal and generalised seizure disorders in both children and adults. An IV formulation of levetiracetam is available for those unable to take oral preparations and appears to have an excellent safety profile including rapid IV use in children [16, 17, 20, 21, 23–26]. In adults, IV infusions of levetiracetam have been well tolerated [27], including at dosages and rates of infusion greater than recommended [28].

Levetiracetam has the following potential advantages when compared to phenytoin for use in CSE. Levetiracetam is easy to administer and can be given as a five-minute infusion into a peripheral IV cannula without the increased risk of serious adverse events (including hypotension, cardiac arrhythmias, extravasation or death). Furthermore, levetiracetam is compatible with both dextrose and normal saline infusion and has limited drug interactions.

On the basis of efficacy from the limited cohort data with levetiracetam, and concerns around low phenytoin efficacy and serious adverse events, IV levetiracetam is being increasingly used as a second line anticonvulsant in CSE in children. However, good quality evidence for IV levetiracetam use in CSE is lacking, and now is an ideal opportunity to compare it to phenytoin, the current recommended standard of care, in the robust environment of a randomised controlled trial (RCT).

## Methods/Design

### Aim

The primary aim of the study is to determine whether IV levetiracetam or IV phenytoin is the better second line treatment for the emergency management of CSE in children. Specifically, we hypothesise that children treated with IV levetiracetam for CSE will do better than children treated with IV phenytoin in terms of time to clinical cessation of seizure activity, need for RSI for on-going seizure management, need for ICU admission, serious adverse events, length of hospital stay, health care costs, and long-term outcome.

### Design

This is a RCT comparing IV levetiracetam with IV phenytoin in children presenting to EDs with CSE who are still seizing after two doses of benzodiazepines. The study will follow the Consolidated Standards of Reporting Trials (CONSORT) guidelines.

### Participants

200 children aged between 3 months and 16 years presenting with CSE to EDs. The study is ongoing with 147 participants enrolled as of April 2017.

### Setting

The study is taking place in 13 EDs in New Zealand and Australia that are members of the Paediatric Research in Emergency Departments International Collaborative (PREDICT), in New Zealand; Kids First Children’s Hospital, Auckland, Waikato Hospital, Hamilton, and Starship Children’s Hospital, Auckland; in Australia; Princess Margaret Hospital for Children, Perth, WA, Women’s and Children’s Hospital, Adelaide, SA, Royal Children’s Hospital, Melbourne, VIC, Monash Medical Centre, Clayton, VIC, Children’s Hospital at Westmead, Sydney, NSW, Sydney Children’s Hospital, Sydney, NSW, John Hunter Hospital, Newcastle, NSW, Gold Coast University Hospital, Southport, QLD, Lady Cilento Children’s Hospital, Brisbane, QLD, and Townsville Hospital, Townsville, QLD. The annual paediatric census of the participating 13 EDs is approximately 500,000. The central site for the study is Starship Children’s Hospital, Auckland, New Zealand.

### Time frame

Three years.

### Interventions

**Fig. 1:**
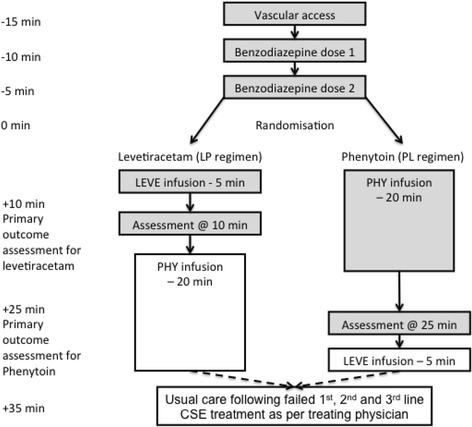
Participant flow for ConSEPT. Management indicated by dashed lines and boxes without fill will only occur if CSE is on-going at that time point. Min = minutes, LP = Levetiracetam phenytoin, PL = Phenytoin levetiracetam, LEVE = levetiracetam, PHY = Phenytoin

Participants will be administered 40 mg/kg IV levetiracetam infusion over 5 min (100 mg/ml levetiracetam (Keppra**®**, UCB Pharma), maximum 3 g, diluted 1:1 with 0.9% sodium chloride to a minimum volume of 10 ml) or 20 mg/kg IV phenytoin infusion over 20 min (50 mg/ml phenytoin (DBL**™** Phenytoin, Hameln Pharmaceuticals) maximum 1 g, diluted 1:4 with 0.9% sodium chloride to a minimum volume of 20 ml). The primary outcome is assessed 5 min following the end of the study intervention infusion. However, if seizures persist the alternative medication will be administered; IV phenytoin infusion if levetiracetam given first (LP regimen), IV levetiracetam if phenytoin given first (PL regimen). See Fig. 1 for study protocol.

### Allocation concealment

A computer generated randomisation code using block randomisation was created by a statistician independent to the study for each site and placed in sequentially numbered opaque, sealed and signed, envelopes for each site by central study pharmacists. Randomisation is stratified by site and age (≤5 years of age and >5 years of age). Stratification by age is utilised to account for the different aetiology of CSE within the paediatric age range.

### Inclusion criteria

Children aged between 3 months and 16 years of age who are currently in CSE, following two doses of benzodiazepines (given by parents, paramedics, or hospital staff), who present to a study ED. CSE is defined as a child who is unresponsive with continuing abnormality of movement (increased tone or jerking) of greater than five minutes duration, or two or more recurrent convulsions without recovery of consciousness between convulsions, or three or more convulsions within the preceding hour, and currently experiencing a convulsion. This definition encompasses the International League Against Epilepsy (ILAE) seizure types of generalised tonic-clonic convulsions, secondarily generalised tonic-clonic convulsions, and complex partial status epilepticus, but not absence, myoclonic, tonic and simple partial status epilepticus.

### Exclusion criteria

Exclusion criteria include previous randomisation, regular phenytoin or levetiracetam use, administration of second line anticonvulsants (phenytoin, levetiracetam, phenobarbitone or paraldehyde) in the last 24 h, a management plan stating refractory to phenytoin, known contraindication or allergy to levetiracetam or phenytoin, CSE due to an obvious major head injury or CSE due to eclampsia in late pregnancy.

### Primary outcome

The primary outcome for the study is clinical cessation of seizure activity five minutes following the completion of the infusion of the study medication (primary efficacy outcome). As study medications have different optimal infusion rates this will be 10 min after starting study infusions in the case of levetiracetam and 25 min after starting study infusions in the case of phenytoin. Blinded confirmation of the primary outcome will occur with the primary outcome assessment being video recorded and assessed by a primary outcome assessment team blinded to treatment allocation.

### Secondary outcomes

Secondary outcomes include: 1.Clinical cessation of seizure activity at two hours following the commencement of the study infusions without the need for further seizure management after the initial agent (levetiracetam or phenytoin); 2.Clinical cessation of seizure activity at two hours following the commencement of the study treatment regimen without the need for RSI or further seizure management (comparison of LP versus PL regimens); 3.Time to clinical seizure cessation from commencement of study treatment regimen; 4.Need for RSI with thiopentone for on-going seizure management after administration of study treatment regimen; 5.ICU admission; 6.Serious adverse events (primary safety outcome) including death, airway complications, and cardiovascular instability (cardiac arrest, arrhythmia and hypotension requiring intervention); 7.Length of Hospital/ICU stay; 8.Health care costs (total costs associated with CSE admission); 9.Seizure status at one month post discharge, or two months post randomisation (whichever is the earliest); 10.Death at one month one post discharge, or two months post randomisation (whichever is the earliest).

### Study process

At all sites patients arriving in clinically diagnosed CSE are assigned an Australasian Triage Category score of 1, as per current procedure, and are immediately taken to the resuscitation area for management. Standard seizure care is initiated in accordance with each site’s clinical practice guidelines, including establishment of IV or intraosseous (IO) access. All clinical practice guidelines, Advanced Life Support Group guideline [4], and the Scottish Intercollegiate Guidelines Network guideline [5], recommend two doses of benzodiazepine prior to initiation of second line seizure medications. For the purposes of the study sites can give their usual benzodiazepine type (diazepam, lorazepam, or midazolam), route (rectal, buccal, oral, intranasal, IV, IO, or intramuscular) and dose. Doses given by parents and/or paramedic staff are regarded as an effective benzodiazepine dose for the purposes of the study. The minimum dose and route of each benzodiazepine is detailed in Table 1.

**Table 1:** Benzodiazepine dosing prior to enrolment in ConSEPT

Benzodiazepine	Route	Minimum dose prior to trial enrolment	Recommended dose*
Diazepam	IV/IO	≥0.1 mg/kg Total dose ≥5 mg	0.25 mg/kg Max 10 mg
	PR	≥0.1 mg/kg Total dose ≥5 mg	0.5 mg/kg Max 10 mg
Midazolam	IV/IO	≥0.1 mg/kg Total dose ≥2 mg	0.15 mg/kg Max 10 mg
	IM	≥0.1 mg/kg Total dose ≥2 mg	0.2 mg/kg Max 10 mg
	Buccal	≥0.1 mg/kg Total dose ≥2 mg	0.5 mg/kg Max 10 mg
	Intranasal	≥0.1 mg/kg Total dose ≥2 mg	0.5 mg/kg Max 10 mg
Lorazepam	IV/IO	≥0.05 mg/kg Total dose ≥2 mg	0.1 mg/kg Max 4 mg

*As per Advanced Paediatric Life Support guidelines (Australia/New Zealand) [4]

*IV* Intravenous, *IO* Intraosseous, *IM* Intramuscular, *PR* Per Rectum

Within each study site’s resuscitation area opaque study boxes for children ≤5 years of age and >5 years of age are stored. When potential patients are moved to the resuscitation areas clinical staff complete a study Clinical Research Form (CRF) addressing inclusion and exclusion criteria for the study. If all inclusion criteria, and no exclusion criteria are present, then clinical staff should open opaque study boxes. Boxes will be opened at the time the second dose of benzodiazepine is given, or on arrival if two doses have already been given, in order to allow nursing staff appropriate time to draw up the infusions of second line anticonvulsant agents. Opaque study boxes contain: An opaque, sealed and signed, envelope containing randomisation allocation and infusions instructions; A Timer; A video device and instructions; Seizure charts; Study information sheet and consent form.

According to the randomisation allocation clinical staff will draw up and administer a levetiracetam (5-min infusion) or phenytoin infusion (20-min infusion) (see Fig. 1, time 0 = start of study infusion). While the first study medication is being administered clinical staff will draw up the alternative study medication.

Five minutes following the completion of study medication infusion a formal assessment of seizure activity will be performed by the most senior treating physician. This assessment is video recorded to allow blinded confirmation of the primary outcome. The participant will be examined for the following: i)Increased tone; ii)Jerking movements (including nystagmoid jerking eye movements); iii)Level of consciousness according to the Alert, Voice, Pain, Unresponsive (AVPU) scale. Continued seizure activity is defined as presence of either increased tone or jerking movements. If seizure activity is present then the alternative study medication is to be infused (phenytoin if levetiracetam given or levetiracetam if phenytoin given). Five minutes following the completion of the second infusion (if required) a formal assessment of seizure activity will again be performed by the most senior treating physician.

If at any stage seizure activity has ceased (as per above definition) the time is recorded and participants will finish the infusion they are currently receiving. No further infusions will be commenced if participants remain seizure free. If seizure activity recommences and participants have only received one study infusion they can be treated with the other medication if this is felt to be appropriate by the treating team.

Clinical or research staff will collect the following data: Demographics; Date of presentation; Date and time of onset of seizure; Benzodiazepine type, dose, route and time given; Highest recorded temperature during resuscitation, at home or with ambulance service; Adverse events occurring prior to starting study medications requiring an intervention (airway repositioning, oral or nasal airway placement, application of positive pressure or ventilation with bag mask, tracheal intubation, fluid bolus, chest compressions, cardiac defibrillation); Adverse events occurring anytime in the first two hours after starting study infusions (in addition to above allergic reaction, IV/IO access tissued, extravasation of IV infusions, purple glove syndrome, any other clinical events deemed significant).

Trained research nurses will visit participants daily while they remain in-patients and contact families one month following discharge collecting the following data: Past medical history; Epilepsy/seizure history; Medication history; Background of presenting event; Family history; Length of stay in hospital/ICU; IV and nasogastric fluids use; Ventilator support; Medications; Seizures; Adverse events; Seizure classification during admission; Neurological investigations.

### Blinded confirmation of primary outcome

In order to increase the robustness of the primary outcome assessment seizure continuation or cessation is video recorded and will be independently assessed by a blinded primary outcome assessment committee (comprising three study physicians, including at least one study ED physician and one study neurologist). At the time of primary outcome assessment the treating team will record the senior treating physician assessing the following: 1.Assessment of tone in lower limbs (i.e. flexion of bilateral ankles for clonus, or flexion of bilateral elbows) - approximately 10 s recording verbally confirming the presence or absence of increased tone; 2.Assessment of jerking movements by recording the hands of the patient (in cases of unilateral seizures the affected side, in cases of predominantly lower limb seizures the lower limbs) – approximately 20 s recording verbally confirming the presence or absence of jerking movements; 3.Assessment of jerking movements by recording the eyes of the patient – approximately 10 s recording verbally confirming the presence or absence of nystagmoid jerking eye movements; 4.Participant study ID.

Prior to the video’s being reviewed by the blinded primary outcome assessment committee they will be reviewed by the study management team and edited so that any part of the video that confirms study medication is removed (i.e. syringe driver with study medication labelled accidentally included in video recording).

### Adverse events

An independent three member Data Monitoring Committee (DMC), comprised of two clinicians each with both emergency medicine and ethics experience, and a biostatistician, has been established. The DMC will receive interim reports every 6 months of adverse events: Episodes of airway repositioning, oral or nasal airway placement, application of positive pressure or ventilation with bag mask, fluid boluses, and extravasation of IV/IO fluids in the first 2 h after starting study medication infusions; Episodes of tracheal intubation in the first 48 h; All episodes of chest compressions, cardiac defibrillation, allergic reactions, or purple glove syndrome.

The following are considered Serious Adverse Events (SAEs): Death; Serious airway complications in the first 24 h, defined as the “unexpected” use of an endotracheal tube, LMA; and cricothyrotomy. “Unexpected” is defined as the use of these interventions when it was not part of a planned RSI following failure of medical management, nor airway support required by a patient who develops a compromised airway secondary to seizure activity or first line CSE medications e.g. benzodiazepines; Cardiovascular instability (cardiac arrest or arrhythmia requiring electrical cardioversion); Any other event that is a life-threatening event. SAEs will be reported to the principal investigator within 24 h, and will be reported to the chair of the DMC within 48 h. The DMC will receive an interim analysis of trial data following the recruitment and follow-up of the first 100 participants. The study will be terminated early if: 1.The DMC, with regards to currently available evidence, following the death or cardiac arrest of a participant due to a study medication, thought that the risks for individual participants outweighed the benefits of continuing the study; 2.The independent DMC, with regards to currently available evidence, following the analysis from the first 100 participants, thought that the risks for individual participants outweighed the benefits of continuing the study.

### Consent and ethical considerations

Due to the life threatening nature of CSE, and the need for urgent timely treatment, it is not possible to gain informed consent prior to randomisation and treatment in this study. Delayed retrospective consent can be sought in New Zealand if consent prior to the intervention is impracticable and/or undesirable [29] and in Australia if prospective consent is not practicable, there is potential benefit to the patient, risk is low, the research has merit and there is no reason to suspect the parents would not give consent [30]. Ethics approval for the study and the accompanying consent process has been granted by the four ethics committees with governance for the 13 study sites. Thus written informed consent to remain in the study is sought from parents and guardians at the earliest possible time after emergency stabilisation of the CSE, i.e. after seizure cessation or seizure termination by RSI and intubation, by either trained research or clinical staff. Data for children whose parents and guardians do not wish for their child to remain in the study is destroyed, apart from demographic data, and will not be available for data analysis.

The use of videos during resuscitation has been standard of care in some of the PREDICT EDs, where they have been used for resuscitation research and found to be acceptable to families [31]. Consent to use the video recordings is a separate item on the consent form i.e. families can take part in the study but not have their child’s video recordings used. If families do not consent to the video recordings these are deleted immediately at the time of consent.

Due to the study being undertaken exclusively in paediatric participants informed consent is not being sought from participants, but only from their parents and guardians.

Two members of the DMC, one in each country, are available to talk with the parent/guardian(s) on request if the parent/guardian(s) have concerns about the consent process.

### Sample size, power and statistical methods

Using pilot data indicating a phenytoin seizure cessation rate of 60% [15] a total of 91 participants will be required to be randomised into each arm for the study to have at least 80% power to detect a total difference in seizure cessation rates between levetiracetam and phenytoin of 20% (alpha = 0.05). The 80% seizure cessation rate for levetiracetam that this study is powered for is at the conservative range of seizure cessation rates reported in retrospective series (75–100%) [19, 21, 22]. To allow for loss to follow-up a total of 100 participants will be randomised into each arm of the study.

Given that the five-year pilot study showed an average of eight possible participants per site per year the study will require three years to complete (8 participants × 13 sites × 3 years = 312). This allows for a third of possible participants to be lost due to exclusions, failure to enrol, or refusal of consent.

Analysis will be by intention to treat. Results from unadjusted comparisons between groups will be reported, together with analyses adjusted for possible imbalances between groups for results with appropriate data distributions. Categorical outcome variables (including the primary outcome) will be compared with chi-squared tests (unadjusted) and logistic regression (adjusted). Continuous outcome variables will be analysed using survival analysis and Cox regression. Continuous outcome variables with skewed distributions will be log-transformed. Continuous variables will be compared with unpaired *t* tests (unadjusted) and linear regression (adjusted). Continuous outcomes variables with skewed distributions after log-transformation will be compared with Mann-Whitney tests. Differences for categorical and unskewed continuous data will be reported as odds ratios (95% confidence interval (CI)) or difference between means (95% CI) respectively. Differences between log-transformed data will be reported as a ratio of geometric means (95% CI). Differences between skewed continuous data will be reported as difference between medians (95% CI). Planned subgroup analysis will be undertaken by focal or generalised onset of CSE, febrile or afebrile CSE, and type of benzodiazepine used. Sensitivity analyses will be undertaken using a modified intention-to-treat dataset (excluding those participants randomised but in whom seizure activity stopped prior to the start of the first study infusion) and a per-protocol dataset.

De-identified data will be collected by trained research nurses, managed using REDCap electronic data capture tools, including data range checks, securely hosted at The University of Auckland, Auckland, New Zealand [32], and analysed using Stata 12 (Statagroup, College Station, Texas, USA). Starship Children’s Hospital, Auckland, New Zealand, is the co-ordinating centre for the study. Study sites will be audited for data collection and management by the co-ordinating centre. No independent audit of data is planned.

A per-protocol analysis of efficacy will be undertaken as a sensitivity analysis. A further sensitivity analysis will be undertaken using the blinded confirmation of the primary outcome data.

## Discussion

### Limitations

The primary outcome does not include electroencephalography (EEG) confirmation of seizure termination. While it is possible that a number of participants may have the “termination of seizure status” misclassified following the study infusion the primary end-point of the study is a pragmatic end-point and reflective of the real clinical world practice and clinical decision points. EEG confirmation of seizure activity is not routinely available in any of the study sites’ EDs, or indeed internationally in EDs.

In addition, the lack of EEG confirmation of seizure activity may possibly result in some pseudo seizures or seizure mimics enrolled in the study. In reality this is very unlikely and if such conditions were to be enrolled the presence of randomisation will make the effect minimal on the overall study results.

Those assessing the primary end-point are not blinded to the assigned intervention group and it is possible that this lack of blinding could introduce bias. As the two study interventions have different optimal infusion times, manufacturing presentations (vials and ampoules), and due to manufacturing technical difficulties related to phenytoin’s high pH we could not instigate a blinded study. However, due to the life threatening nature of CSE it is unlikely that a physician would report that seizure activity had terminated when in fact it had not. Furthermore, the independent video confirmation of the primary outcome assessment will also reduce this possible bias.

### Time line

The study commenced recruitment in March 2015, with the first patient enrolled on the 19th March 2015. Recruitment is expected to finish in 2018. This study is expected to provide robust evidence for second line management of CSE in children.

## Abbreviations

CI: Confidence interval
CNS: Central nervous system
ConSEPT: Convulsive Status Epilepticus Paediatric Trial
CONSORT: Consolidated Standards of Reporting Trials
CRF: Clinical research form
CSE: Convulsive status epilepticus
DMC: Data Monitoring Committee
ED: Emergency department
EEG: Electroencephalography
ICU: Intensive Care Unit
ILAE: International League Against Epilepsy
IO: Intraosseous
IV: Intravenous
LP: Levetiracetam phenytoin
PL: Phenytoin levetiracetam
PREDICT: Paediatric Research in Emergency Departments International Collaborative
RCT: Randomised control trial
RSI: Rapid sequence induction
SAE: Serious adverse event
